# Impact of a Hybrid Preventive Program Combining FIFA 11+ and Customized Neuromuscular Interventions on Lower-Limb Function and Performance in Recreational Mini-Football Players

**DOI:** 10.3390/sports14070259

**Published:** 2026-06-23

**Authors:** Roxana Mihaela Munteanu, Andrei Marian Feier, Bogdan Voicu, Diana Șandru, Arpad Solyom, Tudor Sorin Pop

**Affiliations:** 1 Doctoral School of Medicine and Pharmacy, George Emil Palade University of Medicine, Pharmacy, Science and Technology of Targu Mures, 540139 Targu Mures, Romania; roxana-mihaela.munteanu@umfst.ro (R.M.M.);; 2 Department M2 Functional and Complementary Sciences, Epidemiology, Ethics and Socio-Human Sciences, George Emil Palade University of Medicine, Pharmacy, Science, and Technology of Targu Mures, 540139 Targu Mures, Romania; 3 OKF Medical Center, 540027 Targu Mures, Romania; drbogdanv@gmail.com (B.V.); sandrudiana58@yahoo.com (D.Ș.); arpad.solyom@umfst.ro (A.S.); 4 Department M4 Clinical Sciences, Orthopedics and Traumatology I, George Emil Palade University of Medicine, Pharmacy, Science, and Technology of Targu Mures, 540139 Targu Mures, Romania; 5 Department M3 Clinical Sciences, Orthopedics and Traumatology II, George Emil Palade University of Medicine, Pharmacy, Science, and Technology of Targu Mures, 540139 Targu Mures, Romania

**Keywords:** injury prevention, neuromuscular training, FIFA 11+, performance

## Abstract

**Background**: Recreational mini-football is associated with a high incidence of lower-limb injuries, largely driven by neuromuscular deficits and insufficient exposure to structured preventive training. This study aimed to evaluate the effects of a hybrid injury-prevention program combining the FIFA 11+ protocol with customized neuromuscular interventions on functional performance and injury-related risk factors. **Methods**: Forty male recreational mini-football players were included in a retrospective analysis of data collected during the routine implementation of a 12-week hybrid preventive training program. Participants were allocated to an intervention group (*n* = 20) or control group (*n* = 20) according to routine training practices rather than randomization. The intervention was performed twice weekly. Outcome measures included lower-limb strength (Kineo system), dynamic balance (Y-Balance Test), functional hop performance (Single Hop and Side Hop tests), and agility/change-of-direction ability (Illinois and 505 tests). **Results**: The intervention group demonstrated significantly greater improvements in lower-limb peak force across all muscle groups (all adjusted *p* < 0.001), as well as in single-leg hop, side hop, and agility/change-of-direction performance (all adjusted *p* < 0.001) compared to controls. No significant changes were observed in dynamic balance outcomes. **Conclusions**: A hybrid neuromuscular training program combining FIFA 11+ with customized exercises was associated with improvements in lower-limb strength, hop performance, and agility/change-of-direction ability in recreational mini-football players. These findings suggest that integrating customized exercises into standardized training programs may enhance functional performance and positively influence modifiable factors associated with injury risk.

## 1. Introduction

Football is the most widely practiced sport worldwide, with more than 270 million registered players across professional, amateur, and recreational levels [1,2]. In recent years, recreational forms of football, including mini-football and other small-sided formats, have gained increasing popularity due to their accessibility, social appeal, and recognized health benefits [3,4,5]. Despite being commonly perceived as less demanding than traditional 11-a-side football, recreational mini-football is associated with a substantial injury burden, representing an important public health concern [6].

Mini-football is characterized by high-intensity intermittent activity, frequent accelerations and decelerations, rapid changes of direction, repeated jumping and landing tasks, and play on reduced pitch dimensions. These characteristics result in elevated mechanical loading of the lower extremities and a high prevalence of non-contact injury mechanisms [4,5]. Epidemiological evidence indicates that injury patterns in recreational football closely resemble those observed in traditional football, with the knee, ankle, and hamstring musculature being the most frequently affected anatomical regions [6,7,8,9].

In recreational and amateur football populations, injury incidence during match play remains substantially higher than during training sessions, largely due to the unpredictable and high-intensity nature of game situations [8]. Lower limb injuries account for the majority of time-loss injuries, with knee and ankle sprains and hamstring strains representing more than 50% of all reported cases [9]. Importantly, recreational mini-football players often lack access to structured medical supervision and systematic injury-prevention strategies, which further increases injury risk and prolongs recovery times [10]. Unlike professional football players, recreational mini-football athletes frequently train without access to structured medical support, performance monitoring, or individualized conditioning programs. These limitations may increase the prevalence of modifiable neuromuscular deficits and justify the implementation of accessible preventive strategies in this population.

Non-contact mechanisms are responsible for a large proportion of severe lower extremity injuries in mini-football, particularly those involving the knee joint, such as anterior cruciate ligament (ACL) ruptures [11]. These injuries are commonly sustained during cutting maneuvers, sudden decelerations, pivoting actions, and jump landings—movement patterns that are highly prevalent in small-sided football formats [12,13]. ACL injuries are associated with prolonged rehabilitation, high reinjury rates, and an increased risk of early-onset knee osteoarthritis, even in recreationally active individuals [14].

Altered neuromuscular control, impaired dynamic balance, and deficits in lower limb and trunk strength have been identified as key modifiable risk factors underlying these injury mechanisms [15,16]. Specifically, insufficient eccentric hamstring strength, reduced quadriceps strength, weakness of the hip abductors and adductors, and poor core stability contribute to excessive anterior tibial translation, dynamic knee valgus, and suboptimal load absorption during landing and cutting tasks [17,18]. Such deficits are frequently observed in recreational football players, who often present with inter-limb asymmetries and limited exposure to structured neuromuscular training [19].

To address these risk factors, neuromuscular injury-prevention programs have been widely implemented in football settings. Programs incorporating strength training, balance exercises, plyometrics, and agility drills have demonstrated injury risk reductions of approximately 30–40% in lower limb injuries, with even greater preventive effects reported for knee and ankle injuries [20,21]. These findings highlight the importance of structured preventive strategies, particularly in high-risk recreational populations [22].

One of the most extensively studied injury-prevention strategies in football is the FIFA 11+ program. Originally developed for amateur and recreational football players, the FIFA 11+ consists of a standardized warm-up protocol including exercises targeting core stability, eccentric strengthening of the thigh musculature, proprioception, dynamic balance, and plyometric control [22]. Multiple randomized controlled trials and systematic reviews have confirmed its effectiveness in reducing non-contact injuries across different age groups and playing levels, provided that adequate adherence and supervision are ensured [23,24,25].

Despite its proven efficacy, the standardized nature of the FIFA 11+ program may not sufficiently address individual neuromuscular deficits, biomechanical asymmetries, or sport-specific demands commonly observed in recreational mini-football players [26,27]. Given the heterogeneous physical profiles and training backgrounds of this population, there is growing interest in hybrid preventive approaches that combine standardized warm-up protocols with customized interventions tailored to specific risk factors [28]. Although the FIFA 11+ program has demonstrated substantial effectiveness in improving neuromuscular performance and reducing injury-related risk factors, combining FIFA 11+ with customized interventions may provide additional benefits by addressing common modifiable neuromuscular deficits and movement asymmetries frequently observed in recreational mini-football players and optimizing training adaptations [22,23,24,25,26,27,28].

Therefore, the aim of this study was to implement and evaluate a hybrid injury-prevention program combining the FIFA 11+ protocol with customized lower limb and knee-focused preventive exercises in recreational mini-football players competing at a high competitive amateur level. The program was based on predefined exercise components targeting common modifiable risk factors related to lower-limb injury and was informed by baseline strength, balance, and agility assessments.

It was hypothesized that the hybrid preventive approach would result in superior improvements in lower-limb strength, functional performance, and agility-related parameters compared with usual training routines, while also improving functional and neuromuscular characteristics commonly associated with lower-limb injury risk, rather than directly evaluating injury incidence.

## 2. Materials and Methods

### 2.1. Study Design

This study represents a retrospective analysis of data collected during the routine implementation of a 12-week hybrid preventive training program in recreational mini-football players. Participants were evaluated at baseline (T1) and post-intervention (T2) after 12 weeks, during which the intervention was delivered twice weekly. To assess whether the available sample size provided adequate statistical power, a sample-size adequacy calculation was performed based on knee flexor strength, one of the neuromuscular variables assessed in the dataset, using published data from a comparable soccer intervention study [29]. Based on the reported post-intervention difference in knee flexor strength between groups, a minimum of 18 participants (9 per group) was required to detect a significant difference with 90% power and a two-sided alpha of 0.05. This retrospective study was approved by the Ethics Committee for Scientific Research of the George Emil Palade University of Medicine, Pharmacy, Science and Technology of Târgu Mureș (approval nr. 4040/03 March 2026). Ethics approval was obtained before the retrospective analysis of the existing routine-practice dataset.

### 2.2. Participants

A total of 40 male recreational mini-football players were included and allocated into two groups based on the training strategy applied in routine practice: experimental group (*n* = 20)—hybrid preventive program (FIFA 11+ combined with customized lower-limb and knee-focused exercises); control group (*n* = 20)—usual training routine without the hybrid preventive intervention. Participants in the control group continued their usual mini-football training routine throughout the study period. Regular training sessions consisted of technical and tactical football drills, small-sided games, and match-related activities performed according to the team’s standard practice. No structured neuromuscular injury-risk reduction program or customized corrective exercise program was implemented in the control group. Because group allocation was based on routine practice rather than randomization, selection bias and residual confounding cannot be completely excluded.

Demographic variables recorded included age and body mass index (BMI). Inclusion criteria comprised active participation in recreational mini-football and availability of complete baseline and follow-up testing data. Exclusion criteria included incomplete datasets and any condition preventing participation in testing/training sessions.

**Figure 1:**
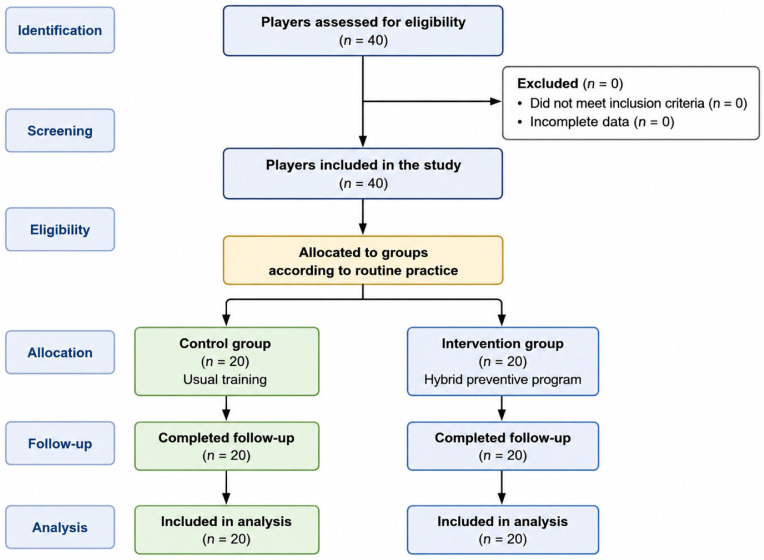
Flow diagram of participant selection, allocation, follow-up, and analysis.

All eligible participants who met the inclusion criteria were included in the analysis. No participants were excluded after enrollment and no loss to follow-up occurred between baseline and post-intervention assessments. A STROBE-style participant flow diagram illustrating eligibility assessment, group allocation, follow-up, and final analysis is presented in Figure 1.

### 2.3. Hybrid Preventive Program (Intervention)

The experimental group performed a 40–45 min hybrid preventive protocol delivered twice per week for 12 weeks, in addition to regular team activity. The protocol combined the standardized FIFA 11+ structure with customized strength, stability, and neuromuscular components targeting modifiable risk factors for knee and lower-limb injuries.

The program was structured into three sections:

1. Dynamic warm-up & running (~8 min): straight-line jogging, lateral/backward running, high knees, and progressive sprints.
2. Strength, stability & prevention (~27–32 min): trunk/core stabilization (plank variations), hip strengthening (glute bridge, clamshells, lateral miniband walks), adductor work (ball squeeze, Copenhagen adduction), eccentric hamstring training (Nordic hamstring), single-leg strength/control (single-leg RDL, single-leg squats with feedback, step-down), landing mechanics (drop jumps), and proprioception tasks (single-leg stance on unstable surface with ball throws).
3. Plyometrics & change of direction (~5 min): controlled vertical jumps, cutting drills (90°/180°), and agility ladder drills.

A detailed exercise list with sets/repetitions and preventive objectives is provided in the protocol documentation.

The detailed structure of the hybrid preventive program is presented in Appendix A (Table A1).

### 2.4. Outcome Measures

All outcome measures were assessed at baseline (T1) and after the 12-week intervention period (T2) using a standardized test battery evaluating lower-limb strength, dynamic balance, functional hop performance, and agility/change-of-direction ability.

#### 2.4.1. Lower-Limb Strength Assessment

Lower-limb muscle strength was assessed bilaterally for the quadriceps, hamstrings, hip abductors, and hip adductors using the Kineo Intelligent Load System (Globus Corporation, Codognè, Italy) in isokinetic concentric mode at an angular velocity of 60°/s. Peak force was recorded in kilograms (kg). The Kineo Intelligent Load System reports peak force values expressed in kilograms (kg), which were used as the outcome measure in the present study. Therefore, the reported outcomes represent Kineo-derived peak force values obtained during isokinetic concentric testing rather than torque values derived from conventional isokinetic dynamometry.

For quadriceps assessment, participants performed leg extension movements in a seated position, completing five maximal repetitions. Hamstring strength was evaluated in standing position through knee flexion movements, while hip abductor and adductor strength were assessed through standing hip abduction and adduction movements, respectively.

For each muscle group, participants performed five repetitions, and the mean value of the five repetitions (expressed in kilograms) was used for statistical analysis. Measurements were recorded separately for the right and left lower limb at both testing sessions.

#### 2.4.2. Dynamic Balance Assessment

Dynamic postural control was assessed using the Y-Balance Test. The test was performed using floor tape markings representing the three reach directions: anterior, posteromedial, and posterolateral. Participants performed the test barefoot while maintaining a single-leg stance on the tested limb and reaching with the contralateral limb in each direction. Three trials were performed for each direction, and the reach distance was recorded. Reach distances were normalized to lower-limb length to account for anthropometric differences between participants.

Y-Balance performance was expressed as a normalized composite score for each limb. The composite score was calculated as the sum of the anterior, posteromedial, and posterolateral reach distances divided by three times the corresponding limb length, multiplied by 100.

#### 2.4.3. Functional Hop Performance

Unilateral lower-limb functional performance was evaluated using the Single-Leg Hop Test.

Participants were instructed to perform a maximal forward hop on a single limb, maintaining balance upon landing. The distance from the starting point to the heel position at landing was measured using a measuring tape. Each participant performed three trials per limb, and the best performance (longest distance) was used for analysis.

#### 2.4.4. Lateral Hopping Capacity

Lateral hopping performance was assessed using the Side Hop Test. Participants performed repeated single-leg lateral hops for 30 s over a 40 cm distance marked on the floor. The total number of correctly executed hops completed within the 30 s interval was recorded.

#### 2.4.5. Agility and Change-of-Direction Performance

Agility performance was assessed using the Illinois Agility Test, performed according to the standardized course configuration. Participants completed three trials, and the best recorded time measured with a manual stopwatch was used for analysis.

Change-of-direction ability was evaluated using the 505 Change-of-Direction Test, following the standard protocol involving a sprint followed by a 180° turn and return. Performance time was recorded using manual timing.

### 2.5. Statistical Analysis

All analyses were performed in Python (version 3.10.2, Python Software Foundation) using the pandas and statsmodels libraries for data management and modelling. Linear mixed-effects models were fitted separately for each outcome to account for the pre–post repeated-measures design, with fixed effects for group, time, and the group × time interaction, and a random intercept for each participant. Age and body mass index were included as covariates in all models. The group × time interaction was interpreted as the intervention effect, reflecting whether the treatment group changed differently over time than the control group. For each mixed-effects model, the treatment effect was defined as the group × time interaction and is reported as an unstandardized regression coefficient (beta) with 95% confidence interval. To aid cross-outcome comparison, a standardized effect size was calculated by dividing the group × time interaction coefficient (β) by the pooled baseline standard deviation of the corresponding outcome. Statistical significance was defined using two-sided tests. Because multiple outcomes were analyzed, *p*-values for the group × time interaction were additionally examined using Benjamini–Hochberg false discovery rate adjustment, with Bonferroni correction performed as a sensitivity analysis. Data are presented as mean ± standard deviation unless otherwise stated.

Outcomes were analyzed using linear mixed-effects models in statsmodels. The reported standard errors correspond to the model-based standard errors of the fixed-effect estimates obtained from the fitted mixed-model results.

To assess the robustness of findings for outcomes with substantial baseline imbalance, a sensitivity analysis was performed using analysis of covariance (ANCOVA) for the Illinois agility test and Y-Balance composite scores. For each outcome, the post-intervention value was entered as the dependent variable, the corresponding baseline value as a covariate, and group, age, and BMI as additional predictors. Heteroskedasticity-consistent standard errors (HC3) were applied. This approach is recommended for pre-post controlled designs with baseline differences (Vickers & Altman, BMJ, 2001) [30].

## 3. Results

### 3.1. Demographics and Descriptive Statistics

A total of N = 40 male athletes were included in the analysis and allocated to control and intervention groups. Group characteristics (age, BMI, training hours, and injury history) are reported as mean ± SD for continuous variables and n (%) for categorical variables (Table 1).

Baseline (pre-intervention) values for strength and functional tests are presented in Table 2. Groups were comparable at baseline (*p* > 0.05) except for Illinois and Y balance tests.

### 3.2. Model Analysis

Linear mixed-effects models were fitted for each outcome with fixed effects for group, time, and the group × time interaction, adjusted for age and BMI, and with participant entered as a random intercept. The group × time interaction was interpreted as the intervention effect, that is, whether the treatment group changed differently over time than the control group.

**Figure 2:**
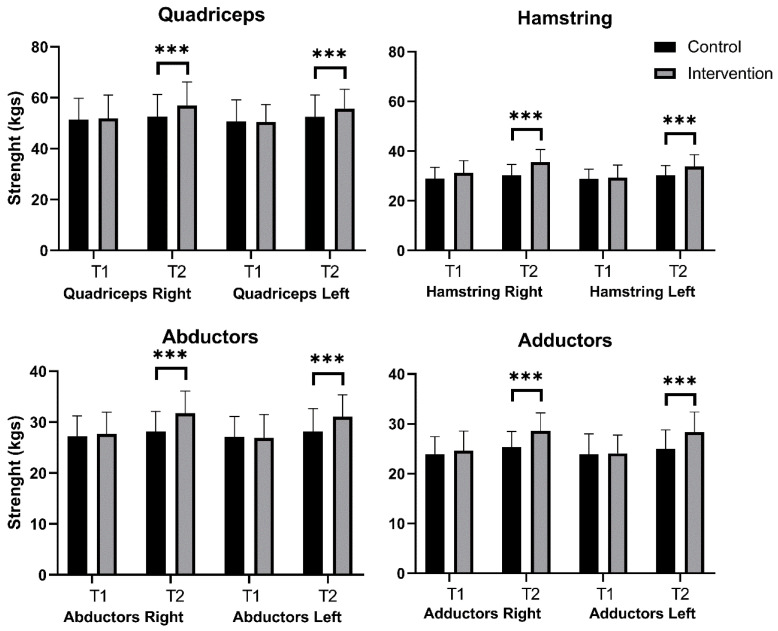
Changes in lower-limb strength outcomes before and after the intervention. Abbreviations: T1 = baseline test; T2 = final test. Data are presented as mean ± SD. *** *p* < 0.001 for the group × time interaction derived from the linear mixed-effects model. Statistical significance refers to group × time interaction effects derived from the linear mixed-effects models and does not represent simple post-intervention between-group comparisons.

Significant group × time interactions were observed for all peak force outcomes, indicating greater improvements in the intervention group than in the control group. These effects were found for quadriceps peak force on the right and left sides, hamstring peak force on the right and left sides, abductors on the right and left sides, and adductors on the right and left sides, with all *p*-values < 0.001. The corresponding interaction coefficients ranged from 2.575 to 3.720, indicating a consistent favorable intervention effect across all strength measures (Table 3). Changes in lower-limb strength outcomes are illustrated in Figure 2.

**Figure 3:**
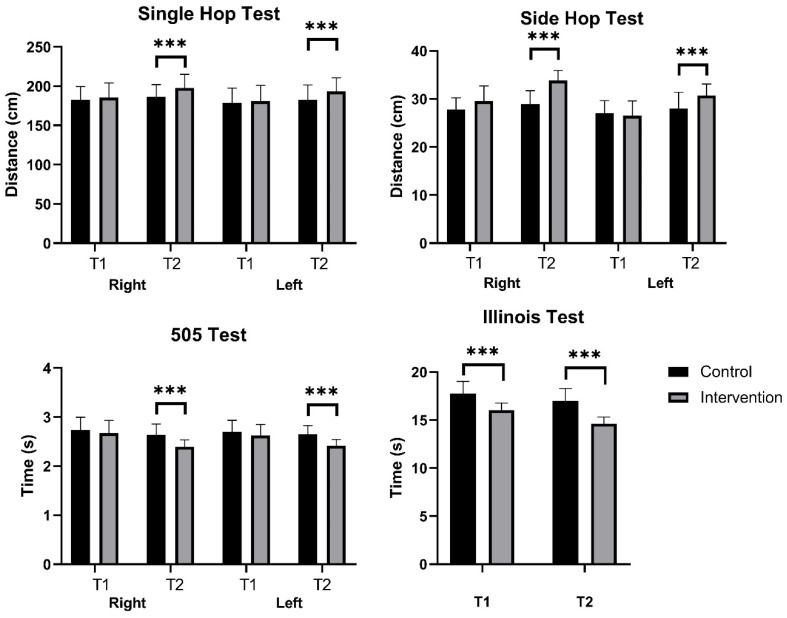
Changes in functional performance outcomes before and after the intervention. Abbreviations: T1 = baseline test; T2 = final test. Data are presented as mean ± SD. *** *p* < 0.001 for the group × time interaction derived from the linear mixed-effects model. Statistical significance refers to group × time interaction effects derived from the linear mixed-effects models and does not represent simple post-intervention between-group comparisons.

Significant intervention effects were also observed for functional performance outcomes. The intervention group showed greater improvement in Single Hop on the right and left sides, Side Hop on the right and left sides, and in both 505 tests compared with the control group, with all *p*-values < 0.001. In addition, Illinois test time decreased significantly more in the intervention group than in the control group (beta = −0.647, SE = 0.125, *p* < 0.001), and both 505 times also showed negative interaction coefficients, indicating superior improvement because lower times reflect better performance. The functional performance outcomes are presented in Figure 3.

By contrast, no significant group × time interaction was observed for Y-Balance composite score on either side. The left composite score showed beta = −0.532, SE = 0.845, *p* = 0.529, and the right composite score showed beta = −0.013, SE = 0.824, *p* = 0.988, indicating no additional intervention-related improvement in dynamic balance compared with the control group.

As sensitivity analyses for multiplicity, both Benjamini–Hochberg false discovery rate and Bonferroni corrections were applied to the group × time interaction *p*-values. Neither approach altered the pattern of statistical significance; all previously significant outcomes remained significant, whereas both Y-Balance composite outcomes remained non-significant.

### 3.3. Sensitivity Analyses

The ANCOVA sensitivity analysis confirmed the primary mixed-model findings (Table S1). After adjustment for baseline performance, age, and BMI, the intervention group demonstrated significantly faster Illinois agility test times at post-intervention compared to the control group (β = −0.80 s, 95% CI: −1.11 to −0.50, *p* < 0.001, R^2^ = 0.939). For the Y-Balance composite scores, the between-group differences remained non-significant after baseline adjustment (left limb: β = +1.10, 95% CI: −1.73 to +3.94, *p* = 0.444; right limb: β = +1.24, 95% CI: −2.57 to +5.06, *p* = 0.523), consistent with the primary analysis.

## 4. Discussion

The present study investigated the effects of a 12-week hybrid injury-prevention program combining the standardized FIFA 11+ protocol with customized neuromuscular and strength exercises in recreational mini-football players. The main findings indicate that the intervention resulted in significant improvements in lower-limb muscle strength, functional hop performance, lateral hopping capacity, and agility/change-of-direction ability. These findings support the effectiveness of hybrid neuromuscular training strategies that integrate standardized preventive programs with customized corrective exercises targeting modifiable risk factors for lower-limb injuries. Interestingly, dynamic balance assessed through the Y-Balance Test did not demonstrate statistically significant improvements following the intervention, suggesting that specific components of postural control may respond differently to hybrid preventive training stimuli.

Lower-limb muscle strength represents one of the most important modifiable factors associated with injury risk in football players, particularly for knee and hamstring injuries [15,16,17,18]. In the present study, significant improvements in quadriceps, hamstring, and hip muscle strength were observed following the 12-week intervention. These findings are consistent with previous research demonstrating that neuromuscular training programs incorporating eccentric strengthening, unilateral exercises, and trunk stabilization can improve lower-limb muscle performance and reduce injury risk [20,21,22,26,27,28]. Daneshjoo et al. reported significant increases in quadriceps and hamstring strength following the implementation of the FIFA 11+ warm-up program in amateur football players, emphasizing the role of structured preventive protocols in improving neuromuscular performance [26]. Similarly, Mendiguchia et al. highlighted the importance of neuromuscular training strategies in addressing common neuromuscular deficits and optimizing lower-limb muscle function [28]. The hybrid approach used in the present study likely enhanced the effectiveness of the standard FIFA 11+ protocol by addressing common neuromuscular deficits identified during baseline assessment.

Significant improvements were also observed in functional performance outcomes, including Single Hop, Side Hop, Illinois Agility, and 505 Change-of-Direction tests. These assessments reflect key components of football performance such as unilateral power, reactive strength, neuromuscular control, and multidirectional movement ability [31,32,33,34,35,36]. The observed improvements are consistent with previous research demonstrating that neuromuscular training interventions incorporating plyometric, strength, and sport-specific movement exercises can enhance hopping performance, landing control, and change-of-direction ability in football players [33,34,35,36,37]. Given the high frequency of cutting, acceleration, deceleration, and multidirectional actions in mini-football, these adaptations may be particularly relevant for improving functional performance in recreational athletes.

Interestingly, dynamic balance assessed through the Y-Balance Test did not show statistically significant improvements following the intervention. This finding suggests that the hybrid program may not have provided a sufficiently specific stimulus to elicit measurable adaptations in dynamic balance. Although participants demonstrated relatively good baseline balance performance, the absence of improvement in both the intervention and control groups indicates that factors other than a potential ceiling effect should be considered [38]. The intervention primarily emphasized strength development, neuromuscular control, and plyometric performance rather than balance-specific training. Previous research suggests that improvements in Y-Balance Test performance are more strongly associated with targeted balance-oriented interventions involving unstable surfaces and progressive proprioceptive challenges [39]. Additionally, some studies indicate that improvements in dynamic balance may require longer intervention durations or higher training frequencies to produce measurable changes in postural control [40].

The overall findings of the present study support the growing body of evidence demonstrating the effectiveness of neuromuscular injury-prevention programs in football populations. The FIFA 11+ program has been widely recognized as one of the most effective structured injury-prevention strategies in amateur football, with several studies reporting reductions of up to 30–50% in lower-limb injury incidence when implemented consistently [23,24,25]. However, recent literature suggests that standardized preventive programs may not fully address individual neuromuscular deficits present in heterogeneous athletic populations [40,41,42]. The hybrid approach used in the present study was designed to overcome this limitation by integrating customized corrective exercises targeting specific muscle groups and movement deficits identified during baseline functional assessments. Such customized strategies have been increasingly recommended in modern sports injury-prevention frameworks, which emphasize the multifactorial nature of injury risk and the need for personalized training interventions [43].

From a broader perspective, the results of this study contribute to the limited body of literature focusing on injury prevention and neuromuscular performance in recreational mini-football players. While most previous studies have investigated elite or professional football populations, recreational athletes represent a particularly relevant group due to their high participation rates and often limited access to structured preventive training programs [6,10]. Therefore, implementing practical and scalable injury-prevention strategies within amateur sports environments remains an important public health priority [44,45].

Although intervention effects were evaluated using linear mixed-effects models that account for repeated measurements over time, baseline differences were present for selected outcomes, particularly the Illinois Agility Test and Y-Balance composite scores. These differences were not only statistically significant but also of potentially meaningful magnitude. Therefore, findings related to these outcomes should be interpreted with caution, as part of the observed between-group differences may reflect baseline imbalance and potential regression-to-the-mean effects rather than intervention-related changes alone.

### 4.1. Practical Implications

Recreational mini-football involves high-intensity intermittent actions that place considerable stress on the lower limbs, while access to individualized conditioning and medical supervision is often limited [4,6].

The hybrid preventive approach, combining the FIFA 11+ protocol with customized neuromuscular exercises, represents a feasible and effective strategy to address these limitations. The improvements observed in performance parameters related to injury risk support its practical applicability.

Furthermore, the program requires minimal equipment and can be easily integrated into routine training sessions, making it suitable for amateur settings. Thus, hybrid neuromuscular training strategies may be effectively implemented by clinicians and coaches to enhance physical performance and address modifiable factors associated with injury risk.

### 4.2. Limitations

Several limitations should be acknowledged when interpreting the findings of the present study. First, the retrospective and non-randomized study design limits the ability to establish causal relationships between the intervention and the observed outcomes. Because participants were allocated according to routine training practices rather than random assignment, selection bias and residual confounding cannot be completely excluded. Outcome assessors were not blinded due to the retrospective nature of the study. However, standardized testing procedures, identical assessment protocols, and the use of the same equipment and testing conditions for all participants were applied to minimize potential observer bias. Future randomized controlled trials would provide stronger evidence regarding the effectiveness of hybrid preventive programs in recreational football populations.

Second, the relatively small sample size may limit the generalizability of the findings. Although the participants represented a homogeneous group of recreational mini-football players, larger and more diverse samples would allow for more robust conclusions and subgroup analyses.

Third, the intervention period lasted twelve weeks, which may not have been sufficient to induce measurable changes in certain neuromuscular parameters such as dynamic balance. Previous studies suggest that balance adaptations may require longer intervention periods or more specific proprioceptive training stimuli [35,36,37].

In addition, baseline differences between groups were present for certain outcome measures, particularly the Illinois Agility Test and Y-Balance composite scores. Although linear mixed-effects models were used to account for repeated measurements and estimate intervention effects, residual influence of baseline imbalance cannot be completely excluded.

Formal reliability analyses were not performed within the present study. Although the selected assessment tools have demonstrated acceptable reliability in previous research, measurement variability cannot be completely excluded.

The Illinois Agility Test and 505 Change-of-Direction Test were timed manually rather than using electronic timing systems, which may have introduced measurement error and reduced measurement precision. In addition, outcome assessors were not blinded, which may have introduced observer bias.

A notable baseline difference was observed in the Illinois agility test between groups (>1.7 s, approximately 1.3–2.3 SDs), which represents a limitation of the randomisation process. A sensitivity ANCOVA adjusting for this baseline difference confirmed that the intervention effect remained significant (β = −0.80 s, *p* < 0.001), supporting the robustness of this finding. Nonetheless, readers should interpret the agility results in light of this pre-existing group difference.

Finally, while injury occurrence was monitored during the study period, the investigation was not specifically powered to detect differences in injury incidence between groups. Future studies should therefore explore whether the observed improvements in neuromuscular performance translate into reduced injury incidence in larger football populations.

## 5. Conclusions

The present study demonstrated that a 12-week hybrid program combining the FIFA 11+ protocol with customized neuromuscular and strength exercises was associated with greater improvements in lower-limb muscle strength, hop performance, and agility/change-of-direction ability compared with routine training alone in recreational mini-football players. These improvements were consistently observed across most strength and functional performance outcomes evaluated.

In contrast, no additional improvements were observed in Y-Balance composite scores, suggesting that the intervention may have been insufficient to produce measurable changes in dynamic balance or that balance-specific adaptations require different training stimuli or longer intervention periods.

Given the retrospective and non-randomized design of the study, the findings should be interpreted with caution. Although the observed improvements involve physical performance parameters commonly associated with lower-limb injury risk, no conclusions can be drawn regarding injury incidence or injury prevention effectiveness. Future prospective randomized studies are needed to confirm these findings and to determine whether improvements in neuromuscular performance translate into reduced injury occurrence in recreational football populations.

## Figures and Tables

**Table 1:** Demographic and training-related data of subjects.

	Control (N = 20)	Intervention (N = 20)	*p*
Age (years)	26.90 ± 3.19	27.45 ± 2.62	0.555
Body-mass index (kg/m^2^)	24.05 ± 2.17	24.08 ± 1.47	0.962
Training exposure (h/12 weeks)	23.6 ± 0.59	23.7 ± 0.80	0.656
Injuries during 12-week intervention, n (%)	0 (0%)	1 (5%)	1.000

**Table 2:** Baseline power and balance performances of athletes before training protocol.

	Control (N = 20)	Intervention (N = 20)	*p* Value
Quadriceps Peak Force–right leg (kg)	51.34 ± 8.44	51.93 ± 9.08	0.833
Quadriceps Peak Force–left leg (kg)	50.76 ± 8.43	50.44 ± 6.93	0.896
Hamstring Peak Force–right leg (kg)	28.93 ± 4.57	31.20 ± 4.95	0.140
Hamstring Peak Force–left leg (kg)	28.90 ± 3.83	29.35 ± 5.00	0.748
Hip Abductors Peak Force–right leg (kg)	27.17 ± 4.03	27.66 ± 4.31	0.712
Hip Abductors Peak Force–left leg (kg)	27.07 ± 4.05	26.93 ± 4.58	0.919
Hip Adductors Peak Force–right leg (kg)	23.94 ± 3.52	24.63 ± 3.92	0.561
Hip Adductors Peak Force–left leg (kg)	23.85 ± 4.13	24.08 ± 3.67	0.853
Single Hop Test–right leg (cm)	182.6 ± 16.88	185.3 ± 18.84	0.642
Single Hop Test–left leg (cm)	178.5 ± 19.17	180.8 ± 20.10	0.713
Side Hop Test–right leg (repetitions)	27.75 ± 2.51	29.55 ± 3.17	0.053
Side Hop Test–left leg (repetitions)	27.05 ± 2.58	26.55 ± 3.07	0.580
505 Test–right leg (s)	2.73 ± 0.27	2.68 ± 0.25	0.512
505 Test–left leg (s)	2.70 ± 0.24	2.63 ± 0.23	0.329
Illinois test (s)	17.76 ± 1.29	16.03 ± 0.74	<0.001
Y Balance composite score Right (%)	74.56 ± 4.62	83.83 ± 4.72	<0.001
Y Balance composite score Left (%)	77.34 ± 3.55	86.44 ± 4.25	<0.001

**Table 3:** Linear mixed-effects model results for intervention effects on strength, functional performance, and balance outcomes.

Test	Unadjusted Beta (95% CI)	SE	Standardized Effect (Beta/SD)	Unadjusted *p* Value	Adjusted *p* Value #	Significant
Quadriceps Peak Force–right leg	3.720 [3.132, 4.308]	0.300	0.424	2.868 × 10^−35^	4.875 × 10^−34^	***
Quadriceps Peak Force–left leg	3.625 [2.832, 4.418]	0.404	0.469	3.136 × 10^−19^	5.331 × 10^−18^	***
Hamstring Peak Force–right leg	3.110 [2.508, 3.712]	0.307	0.653	4.231 × 10^−24^	7.193 × 10^−23^	***
Hamstring Peak Force–left leg	3.015 [2.431, 3.599]	0.298	0.677	4.788 × 10^−24^	8.140 × 10^−23^	***
Hip Abductors Peak Force–right leg	3.130 [2.654, 3.606]	0.243	0.750	4.873 × 10^−38^	8.285 × 10^−37^	***
Hip Abductors Peak Force–left leg	3.000 [2.073, 3.927]	0.473	0.694	2.284 × 10^−10^	3.882 × 10^−9^	***
Hip Adductors Peak Force–right leg	2.575 [2.066, 3.084]	0.260	0.691	3.678 × 10^−23^	6.253 × 10^−22^	***
Hip Adductors Peak Force–left leg	3.170 [2.657, 3.683]	0.262	0.811	8.702 × 10^−34^	1.479 × 10^−32^	***
Single Hop Test–right leg	8.250 [5.214, 11.286]	1.549	0.461	1.004 × 10^−7^	1.707 × 10^−6^	***
Single Hop Test–left leg	8.500 [5.526, 11.474]	1.517	0.433	2.125 × 10^−8^	3.613 × 10^−7^	***
Side Hop Test–right leg	3.150 [2.147, 4.153]	0.512	1.102	7.483 × 10^−10^	1.272 × 10^−8^	***
Side Hop Test–left leg	3.250 [2.362, 4.138]	0.453	1.145	7.418 × 10^−13^	1.261 × 10^−11^	***
Illinois Test	−0.647 [−0.892, −0.402]	0.125	−0.616	2.324 × 10^−7^	3.951 × 10^−6^	***
505 Test–right leg	−0.194 [−0.275, −0.112]	0.042	−0.742	3.391 × 10^−6^	5.765 × 10^−5^	***
505 Test–left leg	−0.156 [−0.226, −0.086]	0.036	−0.676	1.313 × 10^−5^	2.232 × 10^−4^	***
Y Balance composite score–right leg	−0.012 [−1.627, 1.602]	0.824	−0.003	0.988	1.000	ns
Y Balance composite score–left leg	−0.532 [−2.188, 1.124]	0.845	−0.120	0.529	1.000	ns

Legends: SE = model-based standard error obtained from the fitted linear mixed-effects model; *** *p* < 0.001; ns = non-significant; # = Bonferroni-corrected alpha = 0.002941. Standardized effect size = β divided by the pooled baseline standard deviation of the corresponding outcome. Data are presented as fixed-effect estimates from the linear mixed-effects model with model-based standard errors.

## Data Availability

The data presented in this study are available on request from the corresponding author.

## Supplementary Materials

The following supporting information can be downloaded at: https://www.mdpi.com/article/10.3390/sports14070259/s1, Supplementary Table S1: Results of the sensitivity analysis using ANCOVA test.

## Appendix A

**Table A1:** Structure of the Hybrid FIFA 11+ Training Program.

Section	Exercise	Sets/Reps/Duration	Functional Objective
**Part I–Dynamic Warm-up & Running (8 min)**	Jogging (straight line)	2 × 40 m	General warm-up and increased muscle temperature
	Lateral and backward running	2 × 40 m	Coordination and activation of stabilizing muscles
	High knees/running on toes	2 × 40 m	Quadriceps and calf activation
	Progressive sprints	2 × 40 m	Neuromuscular activation and ankle stability
**Part II–Strength, Stability & Neuromuscular Control (27–32 min)**	Plank (progressions)	3 × 60 s	Core stability and lower-limb control
	Side plank	2 × 30 s/side	Lateral core and pelvic stability
	Glute bridge	3 × 12 reps	Glute activation and pelvic control
	Clamshells with miniband	2–3 × 12–15 reps/side	Gluteus medius activation and dynamic valgus control
	Lateral walks with miniband	2 × 10–12 steps/direction	Hip abductor strengthening and pelvic stability
	Ball squeeze between knees (isometric/dynamic)	2 × 8–10 reps × 5 s hold	Adductor activation and hip stability
	Copenhagen Hip Adduction	3 × 8–10 reps/side	Eccentric adductor strengthening
	Forward lunge/Walking lunge	2 × 10 reps/leg	Functional quadriceps strength and dynamic knee control
	Nordic hamstring exercise	2–3 × 5–8 reps	Eccentric hamstring strengthening
	Single-leg Romanian deadlift	2 × 10 reps/leg	Balance, eccentric control, and posterior-chain strengthening
	Single-leg squat with feedback	3 × 8 reps/leg	Dynamic knee alignment and movement control
	Drop jump with controlled landing	3 × 6 reps	Landing mechanics and lower-limb control
	Lateral bounds (single-leg hops)	2 × 8 reps/side	Dynamic knee stability and unilateral power
	Single-leg stance on BOSU + ball throws	2 × 30 s/leg	Proprioception and neuromuscular control
	Step-down from box/stepper	3 × 10 reps/leg	Knee–hip–ankle alignment control
**Part III–Plyometrics & Change of Direction (5 min)**	Vertical jumps with controlled landing	3 × 8 reps	Landing technique and force absorption
	Sprint with changes of direction (90°/180°)	4 × 20 m	Change-of-direction ability and movement control
	Agility ladder drills	2–3 min	Speed, agility, and neuromuscular coordination

Abbreviations: FIFA = Fédération Internationale de Football Association; BOSU = Both Sides Utilized.
