# Supplementary material for: Impact of a Hybrid Preventive Program Combining FIFA 11+ and Customized Neuromuscular Interventions on Lower-Limb Function and Performance in Recreational Mini-Football Players
Source: Sports (Basel). 2026 Jun 23;14(7):259. doi: 10.3390/sports14070259 (PMC13418104; doi:10.3390/sports14070259)
Supplement: Supplementary file 1 [file sports-14-00259-s001.zip › sports-4327724-supplementary.pdf]

Supplementary Table S1. Results of the sensitivity analysis using ANCOVA test

**Outcome**	**β (Intervention vs Control)**	**95% CI**	**p-value**	**R^2**	**N**
Illinois Agility Test (s)	−0.800	−1.105 to −0.495	< 0.001	0.939	40
Y-Balance Composite Left (%)	+1.105	−1.726 to +3.936	0.444	0.859	40
Y-Balance Composite Right (%)	+1.243	−2.571 to +5.056	0.523	0.851	40

ANCOVA model: post-intervention value ~ baseline value + group + age + BMI; HC3-robust standard errors; β = adjusted group difference (Intervention − Control).
